# Antimicrobial activities and mechanism of sturgeon spermary protein extracts against *Escherichia coli*

**DOI:** 10.3389/fnut.2022.1021338

**Published:** 2022-10-03

**Authors:** Ya-Nan Chen, Hai-Lan Li, Jia-Jun Huang, Mei-Jin Li, Tao Liao, Xiao-Yan Zu

**Affiliations:** ^1^Institute of Agricultural Products Processing and Nuclear Agricultural Technology, Hubei Academy of Agricultural Sciences, Wuhan, China; ^2^School of Biological Engineering and Food, Hubei University of Technology, Wuhan, China; ^3^Key Laboratory of Agricultural Products Cold Chain Logistics, Ministry of Agriculture and Rural Affairs, Wuhan, China

**Keywords:** sturgeon spermary, *Escherichia coli*, antibacterial activity, membrane damage, antibacterial mechanisms

## Abstract

This study aimed to evaluate the antimicrobial activities and mechanism of sturgeon spermary protein extracts (SSPE) against *Escherichia coli*. The minimum inhibitory concentration (MIC) and minimum bactericidal concentration (MBC) were determined. Cell structural change was analyzed using scanning electron microscopy-energy dispersive X-ray spectrometry and transmission electron microscope. Moreover, pH, zeta potential, membrane potential, intracellular ATP concentrations and the interaction of SSPE with genomic DNA were analyzed. Results showed that molecular weight of SSPE is 13.4 kDa, the content of basic amino acids is the highest, in which arginine accounts for 73.2%. The MIC and MBC of SSPE for *E. coli* were 0.05 and 5 mg/mL, respectively. After SSPE treatment, cell membrane permeability changes, zeta potential decrease and genomic DNA lysis occurred in *E. coli*, which indicated it exerted bacteriostatic effects either independently or simultaneously by destroying the cell membrane and genomic DNA. These findings indicated that SSPE has potential to be a natural antiseptic.

## Introduction

Given the important biological and economic values of sturgeon caviar, the sturgeon (*Acipenser schrencki*) aquaculture and processing industry has attracted global attention, and the global production of sturgeon reached 102,000 tons in 2017 (1, 2). However, a mass of sturgeon byproducts, such as scales, skins, and viscera, are directly discarded during sturgeon caviar processing. Fish processing wastes serve as potential sources of bioactive substances to enhance their comprehensive utilization level (3). Previous studies reported that bioactive peptides with antioxidant and low-temperature protective functions were isolated from sturgeon skins in Northern China (4). Visceral tissues (swim bladders, intestines, spermary tissues and fish oil, etc.,) accounting for 20–25% of sturgeon body weight are the sources proteins and lipids with high quality (5) that can be used as animal feeds (6) and raw materials to produce lipases and proteases (7). Unfortunately, the processing potentials of spermary tissues, which are rich in various basic amino acids, are seriously neglected.

Some chemical preservatives applied in the food industry, including sodium benzoate, nitrite, and potassium sorbate, have aroused concerns over human health problems (8). Hence, many researchers have dedicated to explore substitutive natural substances (9). Some antimicrobial peptides (AMPs) and proteins have been investigated and commercially applied in the food industry. Extensively existing in vertebrates, invertebrates, plants, and microorganisms, AMPs and proteins (such as protamine, nisin, magainin, and melittin) are a promising substitute for antibiotics (10, 11). Protamine, which exists in mature spermary tissues of fishes, is arginine-rich polycationic basic proteins (12). Their sulfates are clinically used as antiheparin to treat hemorrhage induced by excessive heparin injection (13). Protamine has broad-spectrum antibacterial properties and thus can inhibit the growth of most food-borne bacteria (14). This compound has been used to preserve various kinds of food from candies to fruits and rice and can also be added together with reducing agents to enhance their antiseptic functions in food (15).

Some AMPs and proteins exert their antibacterial effects mainly by acting upon cell membranes. Some peptides can also enter cells by permeating cell membranes or exert their functions by repressing protein folding or enzymatic activity (16), or a combination of two methods generates joint bacteriostatic actions. For instance, MDpep9 separated from the chinese traditional edible larvae of housefly can destroy the cell membrane of *Escherichia coli* and bind to the genomic DNA to inhibit cellular functions and kill cells (17). This study aimed to evaluate the antibacterial activity of protein extracts acquired from sturgeon processing wastes (spermary tissues) against *E. coli* and explore their antibacterial mechanism. The results could elevate the comprehensive utilization level of sturgeons' visceral byproducts and provide a theoretical support for developing novel and promising AMPs as food preservatives.

## Materials and methods

### Extraction of sturgeon spermary protein extracts

Proteins were extracted and modified using a previous method (12). Spermary tissues were segregated from sturgeon processing wastes, soaked in 2% NaHCO_3_ at a material-to-liquid ratio of 1:1 (g/mL) for 30 min, washed once using distilled water, drained, and transferred to a freezing storage at −20°C for later use to remove impurities, such as connective tissues and fats. For RNA removal, the treated spermary tissues were smashed and added into 0.14 mol/L NaCl solution at a material-to-liquid ratio of 1:2 (g/mL), followed by mechanical homogenization for 1 min, stirring in an ice bath for 20 min, standing for 10 min, centrifugation at 6,000 r/min at 0°C for 10 min, and the precipitate was collected. The above steps were repeated twice, and the precipitates were combined. The tissues were then extracted using 1.0 mol/L sulfuric acid at a material-to-liquid ratio of 1:4 (g/mL) for 1 h and centrifuged at 6,000 r/min at 0°C for 10 min. The filter liquor was collected, the above steps were repeated twice, and the three filter liquor samples were combined to obtain a protein extracting solution, which was treated with a nanofiltration membrane equipment and intercepted using 3 kDa spiral wound membrane. The reflux liquid was then precipitated using cold ethanol in a threefold volume and centrifuged at 6,000 r/min and 0°C for 10 min. The filter liquor was discarded, the precipitates were air dried at room temperature, and the solids obtained were named as SSPE.

### SSPE characterization

Protein concentration was determined using Bradford protein concentration determination kit, and protein purity was calculated by the ratio of measured protein concentration to actual protein concentration. The molecular weight of SSPE was characterized *via* Tris-sodium dodecyl sulfate polyacrylamide gel electrophoresis (Tris-SDS-PAGE). The SSPE solution was prepared using 4% concentration gel (pH: 6.8), 15% separation gel (pH: 8.8), and distilled water; blended with 4 X SDS-PAGE loading buffer at a proportion of 4:1; boiled at 100°C for 5 min; and cooled to 20–30°C, followed by sample loading. After SDS-PAGE was finished, the gel was stained using 10% (v/v) acetum containing 0.025% of Coomassie brilliant blueG-250 and then decolored using 10% (v/v) acetum. The hydrolytic amino acid composition and content in SSPE were determined using an automatic amino acid analyzer. In brief, 0.05 g of SSPE samples were weighted and added with 10 mL of 6 mol/L hydrochloric acids to induce hydrolysis for 22 h. The supernatant was separated from the hydrolysate. The sample analysis was performed after being filtered using a 0.22 μm Acrodisc filter.

### Bacterial culture

*E. coli* CCTCC AB93154 was obtained from the China Center for Type Culture Collection (CCTCC, Wuhan, China) and activated by Luria-Bertani Broth (HB0128, Hope Bio-Technology, Qingdao, China). After 24 h of incubation at 37°C, the strain was inoculated into Mueller-Hinton Broth (MHB, HB6231, Hope Bio-Tcehnology, Qingdao, China).

### Determination of minimum inhibitory concentration and minimum bactericidal concentration

Minimum inhibitory concentration (MIC) and minimum bactericidal concentration (MBC) were determined using the conventional broth microdilution method (18, 19) with some modifications. The activated *E. coli* was incubated at 60 r/min and 37°C for 12–16 h to approximately 10^8^ CFU/mL in MHB. Different concentrations of SSPE samples were prepared using sterile MHB and sterilized using a 0.45 μm Acrodisc filter. Afterward, 5.1 mL of the sterile MHB, 0.3 mL of the bacterial culture, and 0.6 mL of SSPE samples with different concentrations (the same volume of MHB was used instead of SSPE as the control) were added into a 15 mL centrifuge tube and incubated at 60 r/min and 37°C for 12 h. The final concentrations of SSPE samples in the 15 mL system were 5, 2, 1, 0.75, 0.5, 0.25, 0.125, 0.05, 0.025, 0.0625, and 0 mg/mL. Afterward, 200 μL of the culture was transferred into each well of a 96-well microtiter plate. OD_600nm_ values were detected at 600 nm using a multifunctional microplate reader (Spark10M, Tecan, Mannedorf, Switzerland). In addition, 100 μL of the culture was spread-plated onto MHB solid medium (1.5% agar was added to MHB). After incubation at 37°C for 12 h, the number of viable bacteria on the plate was counted. MIC was defined as the lowest concentration of SSPE that significantly inhibited the growth of *E. coli* (*p* < 0.05), and MBC was defined as the lowest concentration of SSPE with no visible bacterial growth.

### Scanning electron microscopy with energy dispersive X-ray spectrometry analysis

Scanning electron microscopy (SEM) was used to observe the change of cellular morphologic (20), and energy dispersive X-ray spectrometry (EDS) was used for elemental analysis. As described above, *E. coli* was treated with SSPE samples (the final concentration was 1 mg/mL) and incubated for 6 h and 12 h at 60 r/min and 37°C. The same volume of MHB was used instead of SSPE as the control. After incubation, the suspensions were centrifuged at 6,000 × g for 5 min, washed twice with 0.1 M phosphate buffer solution (PBS, pH 7.4), and resuspended in 2.5% glutaraldehyde fixative for at 12 h. After being washed twice with PBS, the cells were dehydrated in a graded series of ethanol solutions (10, 30, 50, 70, 90, and 100%) for 15 min each and then resuspended in tert-butanol for 30 min at −20°C. Finally, the freeze-dried samples were sputter-coated with gold in an ion coater for 2 min, followed by microscopic examination and analysis by SEM (SU8100, Hitachi, Tokyo, Japan) and EDS (AZtecLive Ultim Max 100, Oxford Instruments, Oxford, UK).

### Transmission electron microscope analysis

Transmission electron microscope (TEM) observation was performed as previously described with some modifications (19). As mentioned in MIC and MBC determination, *E. coli* was treated with SSPE samples (the final concentration was 1 mg/mL) and incubated for 0.5, 6, and 12 h at 60 r/min and 37°C. The same volume of MHB was used instead of SSPE as the control. After incubation, the suspensions were centrifuged at 6,000 × g for 5 min, washed twice with 0.1 M PBS (pH 7.4), and resuspended in 2.5% glutaraldehyde fixative for at 12 h. The cells were then post-fixed for 2 h in 1% OsO_4_ dissolved in PBS at 25°C and washed by PBS three times for 15 min. The cells were then dehydrated in a graded series of ethanol solutions (10, 30, 50, 70, 90, and 100%) for 15 min each, placed in anhydrous acetone for 20 min, transferred to a mixture of anhydrous acetone and epoxy resin (1:1 and 1:3) for 1 h each, and embedded into Epon-812 resin. Ultra-thin sections obtained by cutting these resin blocks into films at a thickness of 500 Å were examined with a TEM (HT7800, Hitachi, Tokyo, Japan).

### Determination of zeta potential and pH

A modified version of (21) protocol was used to determine Zeta potential. As described in determination of MIC and MBC, *E. coli* was treated with SSPE samples (the final concentration was 1 mg/mL) and incubated for 0, 0.5, 3, 6, 9, and 12 h at 60 r/min and 37°C. The bacterial suspensions under different culture times were directly measured by using a potential analyzer (Zetasizer Nano ZS, Malvern Panalytical, UK) and a pH meter (PB-10-C, Sartorius, Cogentin, Germany).

### Determination of membrane potential

Membrane potential was studied according to the method of (22) with some modifications. As described in determination of MIC and MBC, *E. coli* was treated with different concentrations of SSPE samples (the final concentrations were 5, 1, 0.05, 0.01, 0.005, and 0 mg/mL) and incubated for 12 h at 60 r/min and 37°C. The same volume of MHB was used instead of SSPE as the control. After incubation, the suspensions were centrifuged at 6,000 × g for 5 min and washed twice with 0.1 M PBS (pH 7.4). Subsequently, 20 μL of the fluorescent probe bis-(1, 3-dibutylbarbituric acid) trimethine oxonol (5 μM, DiBAC4 (3); Molecular Probes, Solarbio, Beijing, China) and 180 μL of the cell suspensions were added to black opaque 96-well microtiter plates (Nunc, Copenhagen, Denmark). After 15 min of incubation at 37°C in the dark, fluorescence intensity was measured at 490 nm excitation/525 nm emission wavelengths using a multifunctional microplate reader (Spark10M, Tecan, Männedorf, Switzerland).

### Determination of intracellular adenosine triphosphate concentrations

The intracellular ATP concentration of *E. coli* was detected as previously reported (20). As mentioned in MIC and MBC determination, *E. coli* was treated with different concentrations of SSPE samples (the final concentrations were 5, 1, 0.05, 0.01, 0.005, and 0 mg/mL respectively) and incubated for 12 h at 60 r/min and 37°C. The same volume of MHB was used instead of SSPE as the control. After incubation, the suspensions were centrifuged at 6,000 × g for 5 min and washed twice with 0.1 M PBS (pH 7.4). The supernatant was obtained and stored on ice to prevent ATP loss. ATP was measured by using an ATP assay kit (Beyotime Biotechnology, Shanghai, China) in accordance with the manual instructions.

### DNA binding assay

The effect of SSPE on the genomic DNA of *E. coli* was evaluated by agarose gel electrophoresis and ultraviolet (UV) absorption spectroscopy using the method reported by (23) with some modifications. Activated *E. coli* was incubated at 60 r/min and 37°C for 12–16 h to approximately 10^8^ CFU/mL in MHB. The genomic DNA of *E. coli* was extracted using a bacterial genomic DNA extraction kit (DP302, TIANGEN Biotech, Beijing, China). The purity of the extracted genomic DNA was examined by the ratio of optical density (OD) at 260 and 280 nm (OD260/OD280≥1.90). Different concentrations of SSPE samples were prepared using ultrapure water, sterilized using a 0.45 μm Acrodisc filter, and mixed with 3 μL of genomic DNA (200 ng/μL) (The final concentrations of SSPE samples were 5, 1, 0.5, 0.05, 0.005, and 0 mg/mL) at 25°Cfor 15 min. The same volume of sterile ultrapure water was used instead of SSPE as control. After adding 3 μL of 10 × loading buffer, the mixture was subjected to electrophoresis on 8 mg/mL agarose gel. A gel imaging system (Gbox-F3-E, Syngene, Cambridge, UK) was used to observe the gel bands under UV illumination.

In brief, 3 μL of genomic DNA of *E. coli* (200 ng/μL) was mixed with the different concentrations of SSPE samples (the final concentrations of SSPE samples were 1, 0.1, 0.05, 0 mg/mL respectively) at 25°C for 15 min. The same volume of sterile ultrapure water was used instead of SSPE as the control. Subsequently, 2 μL of the culture was added to the Take3 ultramicro detection plate (SN 283324, BioTek, Vermont, USA). UV absorption spectra were measured at 250–330 nm using a multifunctional microplate reader (Synergy^TM^2, BioTek, Vermont, USA).

### Statistical analysis

IBM SPSS software (Version 24.0; SPSS, Inc., Chicago, IL, USA) was used for data processing. All the experiments were performed in triplicate, and the data were presented as mean ± standard deviation. One-way ANOVA with Duncan's test was used to express the significance of differences (*p* < 0.05) between means. All the figures were obtained from Origin (Version 2019b; OriginLab, NOrthampton, USA).

## Results and discussion

### The characterization of SSPE

**Figure 1:**
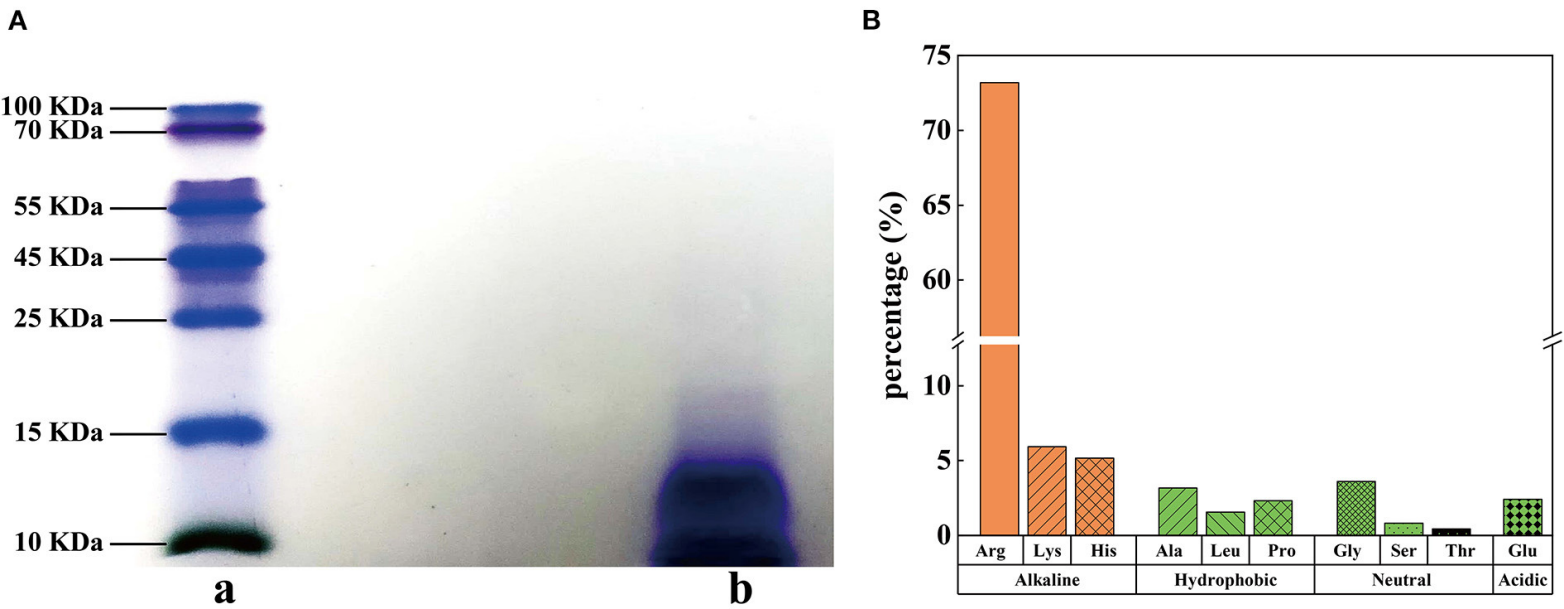
Molecular weight and hydrolytic amino acids in SSPE. **(A)** Tris-SDS-PAGE diagram, a: protein marker, b: SSPE. **(B)** Hydrolytic amino acid composition and contents in SSPE.

Protamine do not have a large molecular weight at 5.5–13.0 kDa (24) and are rich in basic arginine and lysine (25). In this study, the purity of SSPE was determined as 97.7%. The result of Tris-SDS-PAGE revealed that the blue band was below 15 kDa (Figure 1A). According to the migration distance, the molecular weight of SSPE was calculated as about 13.4 kDa. The antibacterial properties of AMPs are believed to be influenced by their structural characteristics, which generally include a high content of positive charges and hydrophobic amino acid residues (26). Hydrolytic amino acid composition analysis showed that SSPE mainly contained three basic amino acids (content: 84.3%), namely, arginine, lysine and histidine, among which, arginine had the highest content accounting for 73.2% (Figure 1B). Hydrophobic amino acids, which might play an important role in maintaining the spatial structure of SSPE, only accounted for 7.1% (Figure 1B).

### Antibacterial activity of SSPE

**Figure 2:**
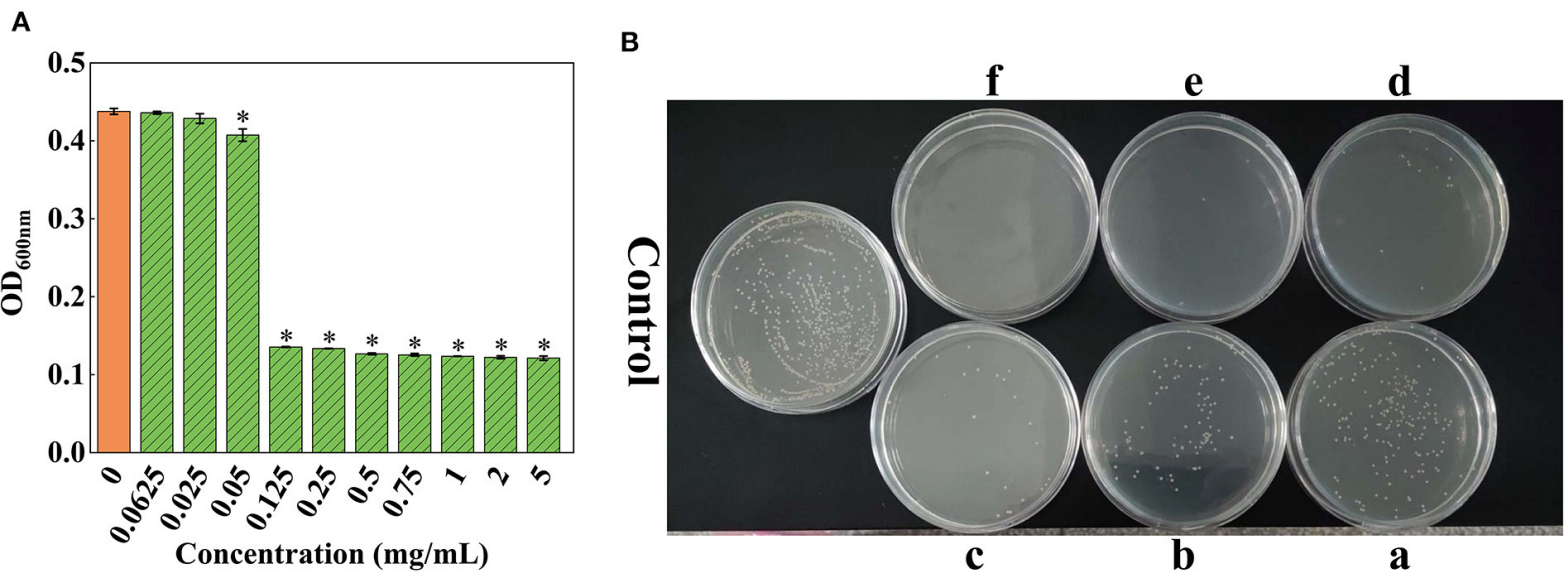
MIC and MBC of SSPE for *E. coli*. **(A)** Effects of solutions with different SSPE concentrations on the optical density (OD) of *E. coli* solution, * indicates significant difference between treatment group and control group in OD (*p* < 0.05). **(B)** Effects of solutions with different SSPE concentrations on the viable count of *E. coli* in MHB solid medium. a: 0.5 mg/mL, b: 0.75 mg/mL, c: 1 mg/mL, d: 2 mg/mL, e: 4 mg/mL, f: 5 mg/mL.

The results showed that OD_600nm_ gradually declined with the reduction in the concentration of SSPE (Figure 2A). When the concentration of SSPE was >0.05 mg/mL, OD_600nm_ significantly dropped compared with that in the control group (*p* < 0.05). When the concentration of SSPE was smaller than 0.05 mg/mL, no significant difference in OD_600nm_ was observed compared with that in the control group (*p* < 0.05). In this case, SSPE generated no inhibitory effect on the growth of *E. coli*. Thus, its MIC was determined as 0.05 mg/mL. MBC was then measured through the bacterial colony count on the agar plate. The results showed that the bacterial colony count on the agar plate gradually decreased with the increase in the concentration of SSPE. When the concentration of SSPE reached 5 mg/mL, almost no colony growth was found on the agar plate (Figure 2B). This indicated that the growth of *E. coli* was completely inhibited by SSPE, and the cells were totally killed in this case. Thus, MBC was determined as 5 mg/mL.

### SEM-EDS analysis

**Figure 3:**
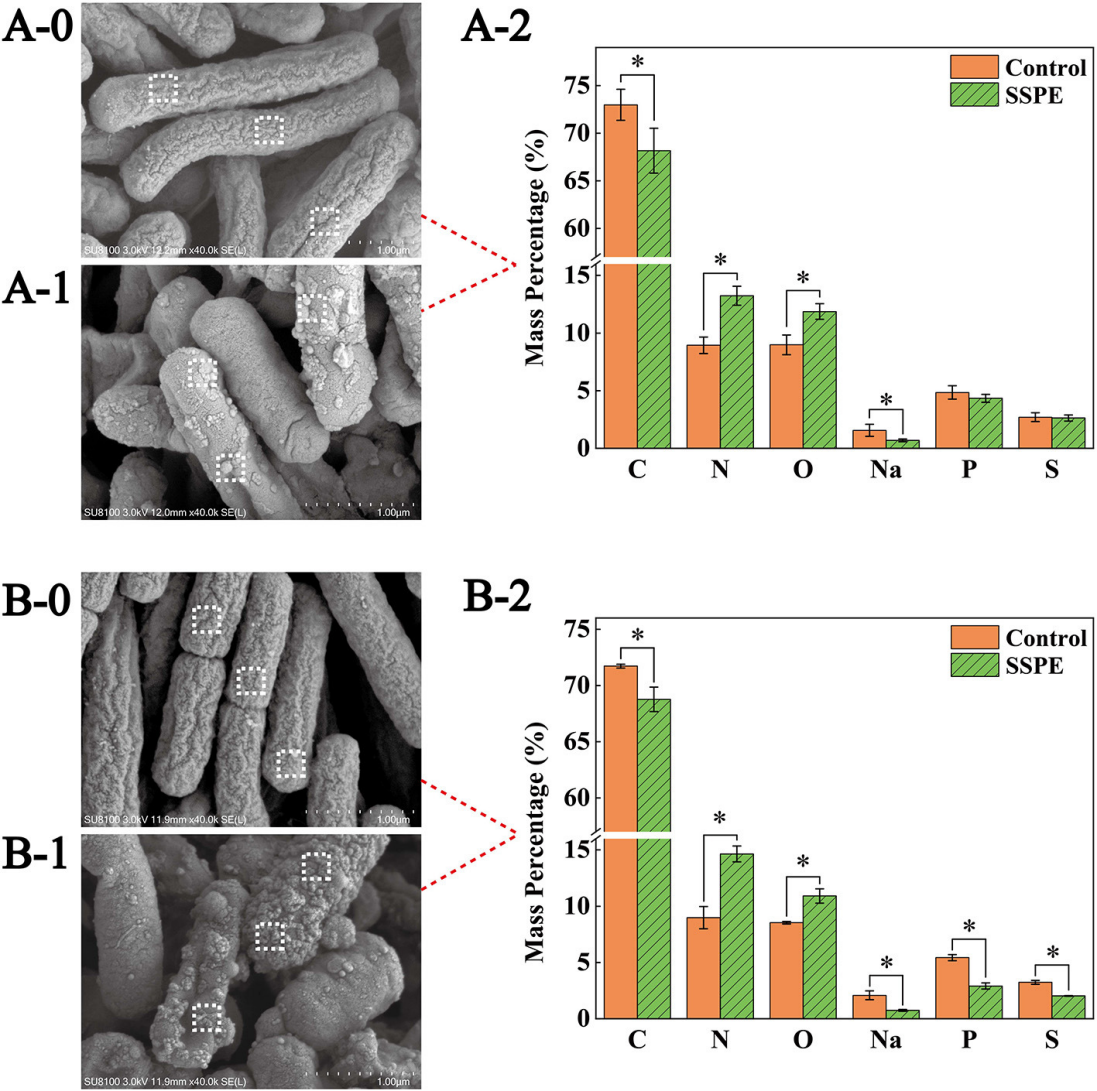
SEM and EDS graphs of *E. coli* under different treatment conditions. Graphs A and B are the SEM and EDS graphs of *E. coli* cultured for 6 and 12 h, respectively. **(A-0)** *E. coli* cultured for 6 h without SSPE treatment, **(A-1)** *E. coli* cultured with 1 mg/mL SSPE for 6 h, **(B-0)** *E. coli* cultured for 12 h without SSPE treatment, **(B-1)** *E. coli* cultured with 1 mg/mL SSPE for 12 h. **(A-2,B-2)** represent the EDS-based spot scanning elemental composition analysis graphs of *E. coli* cultured for 6 and 12 h, respectively. Control: without SSPE treatment. SSPE: treated with 1 mg/mL SSPE. Dotted box denotes the area selected through EDS spot scanning. *indicates significant difference between treatment group and untreated group in element content (*p* < 0.05).

SEM results showed that the *E. coli* untreated with SSPE was rodlike with a full morphology and a clear wrinkled structure on the surface (Figures 3A-0,B-0). For the *E. coli* samples treated with SSPE, particulate matters were adsorbed on the surface, the wrinkled structure changed, and the membrane was subjected to crimping and depression and had fractured. With prolonged culture time, the cell injury was aggravated (Figures 3B-0,B-1). These findings indicated that SSPE might exert its inhibitory and killing effects by changing the cellular morphology of *E. coli* and damaging its cell membrane structure. These actions resembled those of AMPs separated from *Bacillus* secretions in pig intestinal tracts (27). In particular, SSPE induced the formation of a large-scale wrinkle and collapse on the cell surface of *E. coli*, thus inhibiting its growth.

Spot scanning using EDS was performed on the membrane surface to further confirm the particulate matters adsorbed on the cell surface of *E. coli*. Scanning results showed that compared with the control, the mass percentages of C and Na elements on the cell membrane surface dropped significantly (*p* < 0.05)in the treatment (Figures 3A-2), After culturing for 12 h, the mass percentages of P and S elements also declined significantly (*p* < 0.05) in the treatment (Figures 3B-2). However, under different culture time, the mass percentages of N elements and O elements grew significantly (*p* < 0.05) by over 40 and 30%, respectively. A possible reason was that the abundant peptide bonds in SSPE were adsorbed onto the cell membrane surface, leading to the increase in N and O elements. In the meantime, SSPE bound to the cell membrane and posed a potential injury. P and S contents on the cell membrane were reduced because of the volatile lipid bilayer. Studies on antibacterial mechanism revealed the electrostatic attraction between antibacterial substances and cell membrane surface as an important factor (28). ε-Poly-lysine is rich in positive charges and has superior antibacterial activity, causing the aggregation and adhesion of *E. coli* cells, the cell surface showed an irregular shape and experienced shrinkage (29). Owing to its rich amount of positively charged arginine, SSPE bound to the negatively charged phosphate groups on the cell membrane due to electrostatic attraction.

### TEM observation

**Figure 4:**
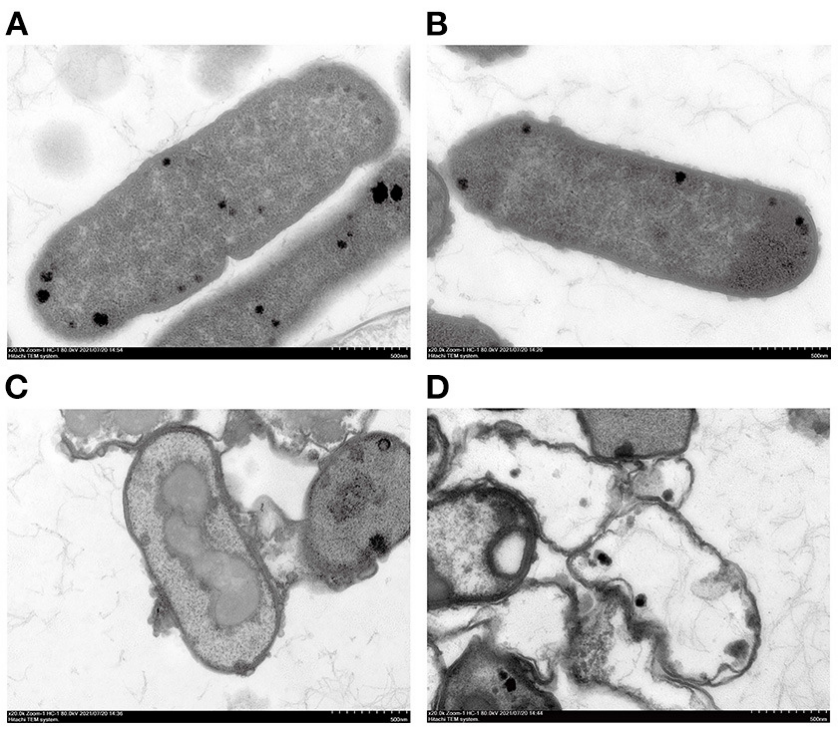
TEM graphs of *E. coli* under different treatment conditions. **(A)** *E. coli* cultured for 12 h without SSPE treatment; **(B)** *E. coli* treated with 1 mg/mL SSPE for 0.5 h; **(C)** *E. coli* cultured for 6 h without SSPE treatment; **(D)** *E. coli* cultured with 1 mg/mL SSPE for 12 h.

TEM results showed that the *E. coli* untreated with SSPE had an intact cell structure, and the intracellular dark part represented cellular contents (Figure 4A). However, the cell structure of *E. coli* treated with SSPE showed substantial changes. In particular, the bacterial capsular layer was dissolved, and irregularly shaped substances adhered to the cell membrane surface (Figure 4B). Liu H et al. (30) also reported similar findings and discovered that chitosan could destroy the outer membrane of *E. coli*. Additional dentate layer was covered on the outer membrane, but the inner membrane was almost not influenced. The substances on the outer membrane surface were speculated as either irregular shapes formed in the gradual dissolution of capsular layer or the SSPE that has entered the cells by some means and resembled the function of cell-penetrating peptides that could selectively permeate into the cell membrane (31). Meanwhile, the clear cytoplasmic region differed in the cells with and without SSPE treatment (Figures 4C,D). After SSPE treatment, the cell membrane structure of *E. coli* ruptured, the cellular contents leaked, vacuoles were formed, and the cells finally disintegrated and died. A study on the inhibitory effect of cinnamon oil on *E. coli* observed a similar phenomenon *via* TEM (19), but some substances might show different phenomena. For instance, Previous study found that the cell membrane surface of *E. coli* treated with chitosan was covered by many vacuolar structures that are possibly caused by the destruction of the outer membrane screen (32).

### Zeta potential and pH

**Figure 5:**
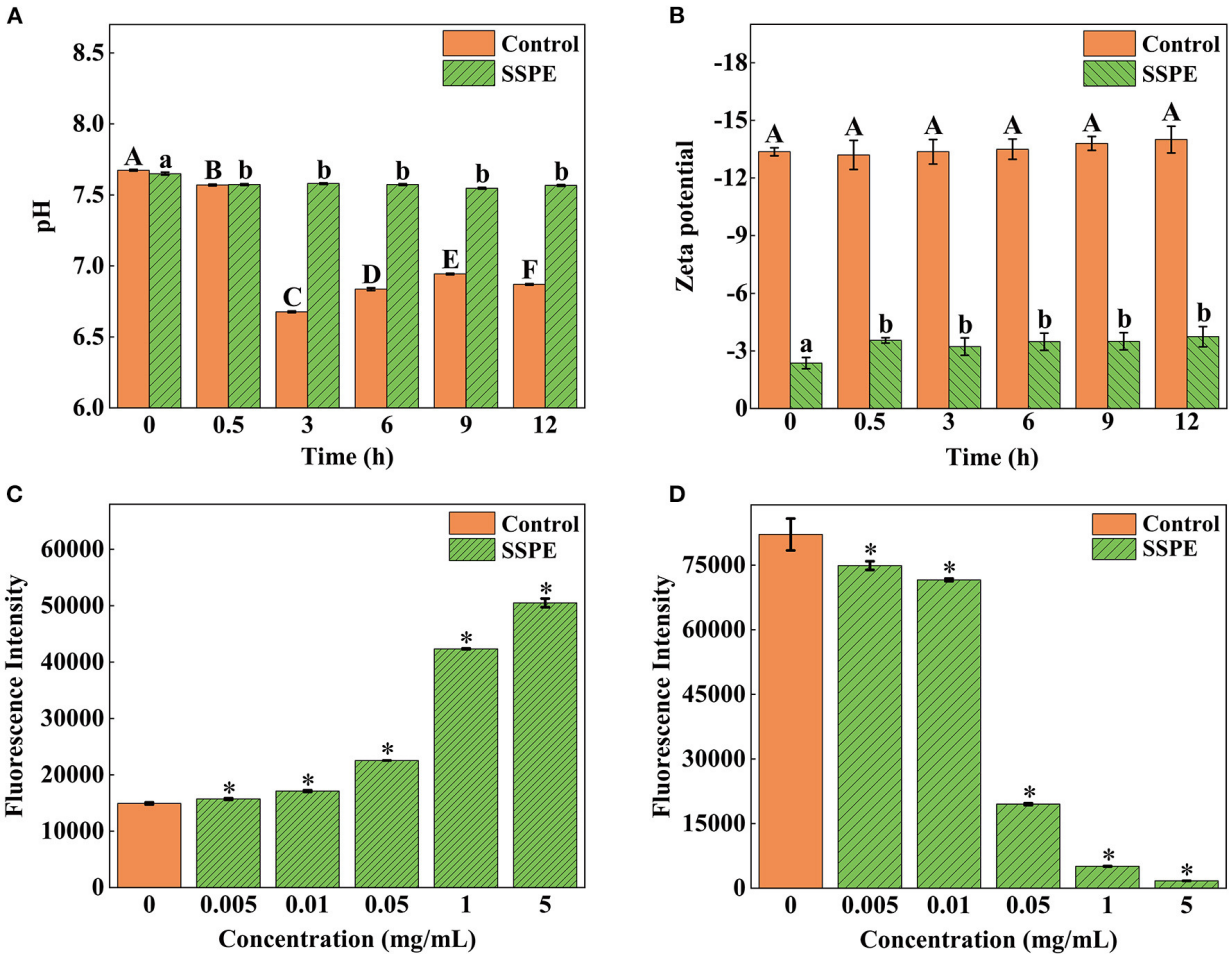
Effects of SSPE on the pH **(A)**, zeta potential **(B)**, membrane potential **(C)** and intracellular ATP concentration **(D)** of *E. coli*. Control: untreated with SSPE; SSPE: treated with 1 mg/mL SSPE. Different capital letters and lowercase letters indicate that the results are significantly different in the same group at different time (*p* < 0.05); *indicates significant difference between treatment group and untreated group (control group) in fluorescence intensity (*p* < 0.05).

A low absolute value of surface potential, usually characterized by zeta potential, results in easy bacterial coagulation and adhesion and a high system stability (33). *E. coli* cells gradually generate some acidic metabolites during growth and metabolic processes. Thus, the pH in the control group continuously declined. Nevertheless, the system pH was elevated at 0.5 h after SSPE treatment. No significant difference (*p* > 0.05) in pH was found later on and its value stabilized at around 7.6 (Figure 5A). This trend occurred possibly because SSPE influenced the synthesis of some metabolites in *E. coli* or neutralized some acidic metabolites to keep a stable system pH. Meanwhile, the absolute value of zeta potential in the system untreated with SSPE was stabilized at about 13.5. After SSPE treatment, the absolute value of zeta potential decreased and was stabilized at 3.4 after 0.5 h (Figure 5B). A possible reason was that SSPE, which was rich in arginine, carried positive charges and bound to negatively charged phosphate groups on the cell membrane of *E. coli*. As a result, bacterial coagulation occurred, and system charges were reduced, leading to the reduction in system zeta potential. Similar to these study results, positively charged lauryl-poly-L-lysine can neutralize the negative charges carried out by negative groups, such as lipoteichoic acid and peptidoglycan, on the cell membrane surface (34).

### Membrane potential

The potential change of *E. coli* was determined using voltage-sensitive specific fluorescent dye DiBAC4 (3). Although it cannot generate any fluorescence itself, *E. coli* would emit fluorescence when bound to depolarized cells or intracellular proteins (35). The results showed that the fluorescence intensity of *E. coli* treated with SSPE was significantly higher than that of the untreated group (*p* < 0.05) and continued to grow with the increase in the concentration of SSPE (Figure 5C). The depolarization of membrane potential, a sign of membrane injury, is mainly related to the release of K^+^ or other ions (36). The above finding revealed that SSPE reduced the membrane potential and induced the depolarization of the cell membrane possibly by changing the membrane permeability, which resulted in K^+^ outflow. As a result, the potential in the membrane became increasingly negative. In addition, SSPE might directly destroyed the cell membrane. According to similar studies, syringic acid can damage the cell membrane of *Cronobacter sakazaki* by the depolarization of the cell membrane (22).

### Intracellular ATP concentration

In ATP bioluminescence method, fluorescein is catalyzed to generate fluorescence according to the reaction principle between firefly luciferase and its substrate ATP, the determined ATP concentration is in a direct proportion to the fluorescence intensity (20). The results showed that the fluorescence intensity of *E. coli* treated with SSPE was significantly lower than that of the control group (*p* < 0.05) and gradually declined with prolonged culture time (Figure 5D).

The ATP level of the intact cells was stable, and the intracellular ATP concentrations might be changed by destroying the intracellular stability and intactness (37). This finding reflected that SSPE reduced the intracellular ATP concentration. The leakage or rapid exhaustion of intracellular ATP might be due to the change in the permeability of cell membrane, membrane injury, or the acceleration of ATP hydrolysis (38). Previous reported that essential oil could reduce the intracellular ATP content of *Lister monocytogenes* and exert the antibacterial effect by inducing cellular leakage (39). Different studies reported that the antifungal activity of salmon spermary proteins may be ascribed to the leakage of ATP and the generation of reactive oxygen species, revealing that these proteins may be internalized through energy dependence mechanism instead of endocytosis (40).

### *In vitro* DNA binding test

**Figure 6:**
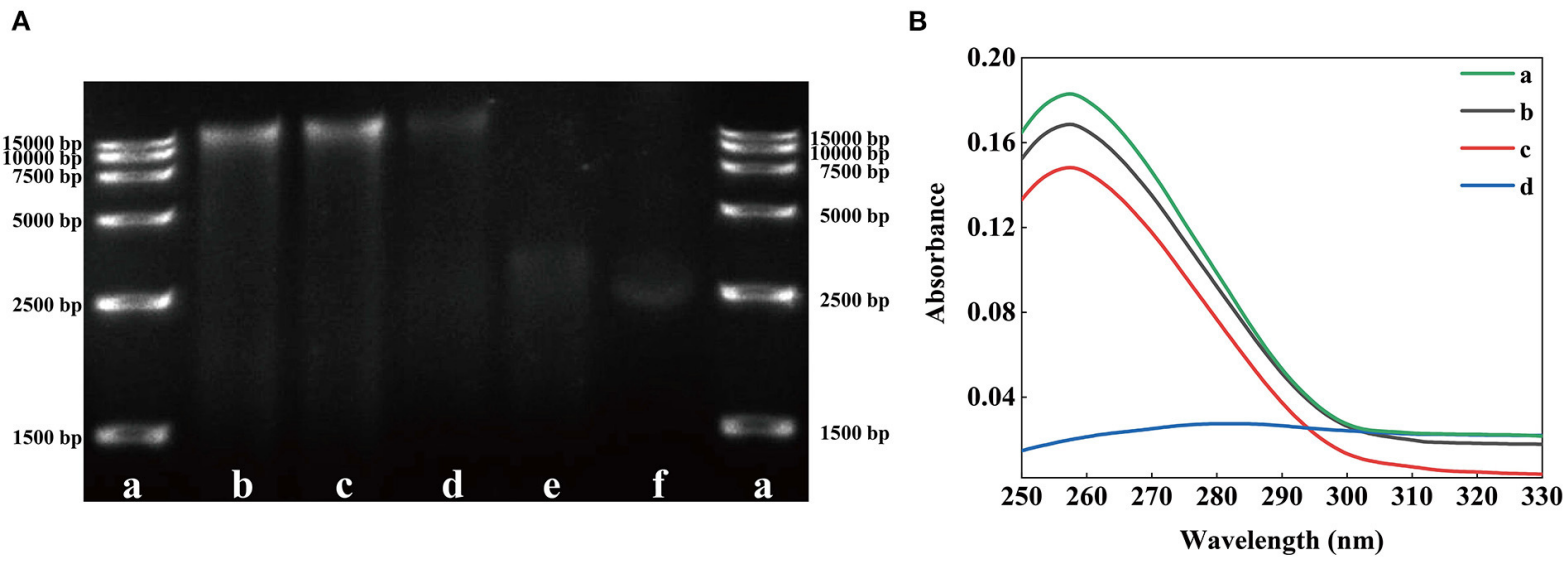
Effects of solutions with different SSPE concentrations on the genomic DNA of *E. coli*. **(A)** Gel electrophoretic band analysis, a: DNA Marker, b: 0 mg/mL, c: 0.05 mg/mL, d: 0.5 mg/mL, e: 1 mg/mL, f: 5 mg/mL. **(B)** UV spectrum of interaction between solutions with different SSPE concentrations and *E. coli* genomic DNA. a: 0 mg/mL, b: 0.05 mg/mL, c: 0.1 mg/mL, d: 1 mg/mL.

Some AMPs destroy the key intracellular components of microorganisms, such as proteins, enzymes, RNA, and DNA, to damage their cell membrane structure (41). TEM results revealed that SSPE might enter cells. However, whether SSPE could bind to key intracellular substances could not be proven only through the membrane potential and intracellular ATP concentration. Therefore, the influence of SSPE on the genomic DNA of *E. coli* was detected through *in vitro* DNA binding test. The DNA electrophoretogram of the control showed clear and bright DNA bands, but showed darkened or disappeared DNA bands after SSPE treatment. The higher SSPE concentration, the darker the band corresponding to 15,000 bp. The bands at 15,000 bp under 1 and 5 mg/mL almost completely disappeared (Figure 6A). UV absorption spectrum reflected that a hypochromic effect was generated due to SSPE–DNA binding. Under SSPE treatment at a concentration of 1 mg/mL, the absorbance value decreased evidently between 250–290 nm, and there was no maximum absorption peak at 260 nm (Figure 6B). This finding confirmed that SSPE exerted a destructive effect on the genomic DNA of *E. coli*, and this ability was directly proportional to the concentration of SSPE. Related studies reflected that some natural antimicrobial substances can disturb important cellular functions by binding to the genomic DNA (18, 23, 42). In this case, SSPE might bind to the genomic DNA after penetrating the cell membrane, disturb the synthesis of related proteins by disintegrating the genomic DNA, and further inhibit or kill the cells.

## Conclusions

SSPE extracted from sturgeon spermary could significantly inhibit the growth of *E. coli*. SSPE is rich in arginine, which could be adsorbed to cell membrane through electrostatic attraction, resulting in structural damage of cell membrane of *E. coli*. Moreover, SSPE changed cell membranes permeability of *E. coli*, and might kill cells by binding to and destroying the genomic DNA after penetrating the cell membrane. Therefore, SSPE might inhibit the growth of *E. coli* independently or simultaneously through the aforementioned two mechanisms. This study shown that sturgeon processing wastes have a potential application in the development of antimicrobial peptides and preservatives.

## Data availability statement

The raw data supporting the conclusions of this article will be made available by the authors, without undue reservation.

## Author contributions

YNC and HLL: methodology, investigation, validation, data curation, and writing-original draft. JJH: data curation, validation, and formal analysis. MJL: data curation and validation. TL: project administration, conceptualization, and resources. XYZ: writing-review and editing, methodology, resources, and funding acquisition. All authors contributed to the article and approved the submitted version.

## Funding

This work was supported by the National Key Research and Development Program of China (2019YFD0902000) and the Key R&D Program of Hubei Province (2022BBA0011).

## Conflict of interest

The authors declare that the research was conducted in the absence of any commercial or financial relationships that could be construed as a potential conflict of interest.

## Publisher's note

All claims expressed in this article are solely those of the authors and do not necessarily represent those of their affiliated organizations, or those of the publisher, the editors and the reviewers. Any product that may be evaluated in this article, or claim that may be made by its manufacturer, is not guaranteed or endorsed by the publisher.
